# Centromeric Barrier Disruption Leads to Mitotic Defects in *Schizosaccharomyces pombe*

**DOI:** 10.1534/g3.114.010397

**Published:** 2014-02-13

**Authors:** Terilyn L. Gaither, Stephanie L. Merrett, Matthew J. Pun, Kristin C. Scott

**Affiliations:** * Institute for Genome Sciences and Policy, Duke University, DUMC 3382, Durham, North Carolina 27708, and; † Department of Molecular Genetics and Microbiology, Duke University Medical Center, Durham, North Carolina 27710

**Keywords:** centromere, genome instability, chromatin, CENP-A, barrier

## Abstract

Centromeres are *cis*-acting chromosomal domains that direct kinetochore formation, enabling faithful chromosome segregation and preserving genome stability. The centromeres of most eukaryotic organisms are structurally complex, composed of nonoverlapping, structurally and functionally distinct chromatin subdomains, including the specialized core chromatin that underlies the kinetochore and pericentromeric heterochromatin. The genomic and epigenetic features that specify and preserve the adjacent chromatin subdomains critical to centromere identity are currently unknown. Here we demonstrate that chromatin barriers regulate this process in *Schizosaccharomyces pombe*. Reduced fitness and mitotic chromosome segregation defects occur in strains that carry exogenous DNA inserted at centromere 1 chromatin barriers. Abnormal phenotypes are accompanied by changes in the structural integrity of both the centromeric core chromatin domain, containing the conserved CENP-A^Cnp1^ protein, and the flanking pericentric heterochromatin domain. Barrier mutant cells can revert to wild-type growth and centromere structure at a high frequency after the spontaneous excision of integrated exogenous DNA. Our results reveal a previously undemonstrated role for chromatin barriers in chromosome segregation and in the prevention of genome instability.

Centromeres are unique loci that direct chromosome segregation during mitosis and meiosis. Mammalian centromeres encompass hundreds to thousands of kilobases of repetitive arrays that assemble into structurally and functionally distinct chromatin domains. For example, centromeric core chromatin is enriched in atypical nucleosomes in which histone H3 is replaced by the evolutionarily conserved centromere-specific histone H3 variant CENP-A. Core chromatin is the structural foundation of the three-dimensional kinetochore, a multiprotein complex that links the chromosome to the mitotic spindle during cell division. Epigenetic mechanisms are involved in determining the genomic location of CENP-A deposition because centromere-associated DNA sequences vary among organisms, among chromosomes of a single organism, and between individuals of the same organism (Henikoff and Furuyama 2010). A second centromeric chromatin domain, pericentric heterochromatin, is characterized by the presence of the conserved heterochromatin protein 1 and nucleosomes di- or trimethylated at lysines 9 and/or 27 of histone H3 (Verdaasdonk and Bloom 2011). Pericentric heterochromatin may provide tension and/or rigidity at centromeres during cell division (Black *et al.* 2004) and is functionally required for chromosome cohesion in some organisms (Bernard *et al.* 2001). In addition to being structurally and functionally distinct, core and pericentric heterochromatin domains are also spatially distinct. Linear chromatin fibers of centromeric loci appear as alternating, nonoverlapping bocks of core and pericentric heterochromatin domains (Blower *et al.* 2002; Sullivan and Karpen 2004; Dunleavy *et al.* 2011). The specification and preservation of discrete chromatin subdomains is closely linked with centromere activity and genome stability. For example, some malignant cells are characterized by an increase in the size of the core chromatin domain and a reduction in pericentric heterochromatin marks (Sullivan *et al.* 2011). Similarly, many tumors that display aberrant cell division and genome instability are associated with aberrant histone modifications (Nakano *et al.* 2008; Bergmann *et al.* 2011; Slee *et al.* 2012). Defects in meiotic chromosome segregation also are correlated with changes in subdomain size and integrity (Scott *et al.* 2006).

The chromatin domain organization of fission yeast *Schizosaccharomyces pombe* (*S. pombe*) centromeres is analogous to that of multicellular, more complex eukaryotes (Kniola *et al.* 2001; Appelgren *et al.* 2003). *S. pombe* centromeres are arranged linearly as a single ~11- to 15-kb region of CENP-A^Cnp1^-containing core chromatin flanked by ~12- to 20-kb domains of pericentric heterochromatin (Figure 1). In contrast to uniform repetitive DNA arrays present at mammalian centromeres, the fission yeast centromeric sequences are arranged in as inverted repeats (including the *otr* and *imr* sub repeat elements) surrounding a central core (*cnt*) element (Pidoux and Allshire 2004). The tandem and inverted arrays of *otr* repeats are organized into pericentric heterochromatin enriched in H3K9me2/3 and the heterochromatin protein 1 homolog, Swi6 (Cam *et al.* 2005). The *cnt* and most of the *imr* repeat incorporate both canonical nucleosomes and the specialized CENP-A^Cnp1^−containing nucleosomes (Partridge *et al.* 2000; Takahashi *et al.* 2000; Cam *et al.* 2005). At the intersection of these structurally discrete chromatin domains are pairs or clusters of *imr*-embedded nonessential transfer RNA genes (tDNAs) (Takahashi *et al.* 1991; Cam *et al.* 2005). The tDNA^Ala^ at centromere 1 (cen1) is a known heterochromatin barrier (Scott *et al.* 2006, 2007), defined operationally as a DNA sequence that restricts the assembly of heterochromatin to specific regions of the genome (Sun and Elgin 1999). Barriers, like insulator elements present in multicellular eukaryotes, protect genes from position-effects originating from the surrounding chromatin environment (Barkess and West 2012).

**Figure 1:**
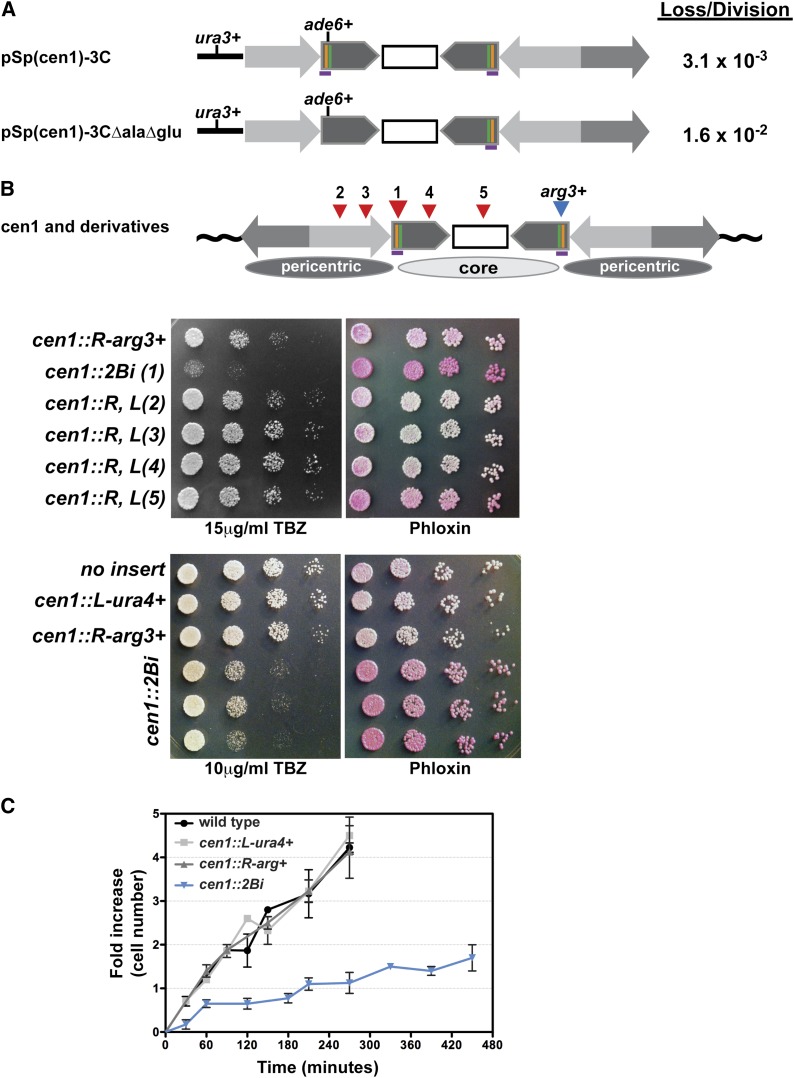
The *cen1*::*2Bi* mutant is defective in viability, growth, and chromosome segregation. (A) The structure of minichromosome pSp(cen1)-3C (Hahnenberger *et al.* 1989, 1991) and derivative pSp(cen1)-3CΔalaΔglu. The cen1 barriers (purple horizontal line) include tDNA^Ala^ (orange vertical line) and tDNA^Glu^ (green vertical line). (B) The structure of centromere 1 (cen1) and derivatives. Reporter gene insert positions are shown as inverted triangles. Core (light gray) and pericentric heterochromatin (dark gray) chromatin domains are illustrated by ovals. Ten-fold dilutions of the indicated strains were spotted onto YES containing TBZ or Phloxine B and grown at 32°. The number in parentheses indicates the position of the *ura4^+^* reporter gene as indicated in (A). Strain *cen1*::*L-ura4^+^* is described in (Scott *et al.* 2006), and contains the *ura4^+^* gene inserted into the cen1 barrier on the left of cen1. (C) Growth kinetics of wild-type, single-insert control and cen1::2Bi strains in selective media at 32°.

In this report we demonstrate that the preservation of both core and pericentric heterochromatin domain integrity requires centromeric chromatin barriers. After insertional mutagenesis of both chromatin barriers flanking the cen1 core domain, cells display reduced fitness and chromosome segregation defects. Mutant phenotypes are characterized by changes in both the density of both CENP-A^Cnp1^−containing nucleosomes and enrichment of H3K9me3 modifications. These data demonstrate that, in addition to blocking the local spread of pericentric heterochromatin, chromatin barriers also contribute to the maintenance of centromere identity, accurate chromosome segregation and the preservation of genome stability.

## Materials and Methods

### Fission yeast methods and strain construction

The genotypes for *S. pombe* strains used in this study are listed in Supporting Information, Table S1. Media were prepared according to standard procedures (Moreno *et al.* 1991). pCen1-3CΔalaΔglu was constructed by transforming pCen1-3C−carrying strain KFY 503 (a gift from Louise Clarke) with a *Bam*HI/ *Hin*cII fragment from plasmid SM636. SM636 was engineered by modification of plasmid SM353 with primers 5055/5056 and 5057/5058 by site-directed mutagenesis as in Scott *et al.* (2006).

The *arg3^+^* gene was amplified as a 1.7-kb *Bam*HI fragment with primers 47332339 and 47332340. Plasmid SM353 (Scott *et al.* 2006) was modified by changing the *Hin*dIII site to a *Bgl*II site and ligated to the *arg3^+^*-containing fragment. The resultant plasmid was digested with *Bam*HI and *Hin*cII, the insert was gel purified and used for transformation with host strain KFY 1400, resulting in *cen1R-arg3^+^* strains. Three independent transformed strains were established, which were confirmed by polymerase chain reaction (PCR) and Southern analysis to confirm the integrity of the locus. Strains were crossed 2−3 times to the host strain before further analyses.

To make strains with two centromeric reporter genes, *cen1*::*R-arg3^+^* was crossed to *cen1*::*L-ura4^+^* derivatives (Scott *et al.* 2006). At least three independent transformed strains were established from each construct. No gross chromosomal rearrangements were detected by Southern Blot in cen1::2Bi strains. Sequencing of DNA ~13 kb between reporter genes and ~1 kb distal to the reporter gene insertion sites in cen1::2Bi strains revealed no sequence alterations when compared with the *S. pombe* standard genome (Wood *et al.* 2002).

### Spotting assays and analysis of growth kinetics

Cells grown to log phase at 32° in Pombe Glutamate Medium (PMG) media under the appropriate selection were spotted in 10-fold serial dilutions and incubated at 32° for 3 d before they were photographed. To determine the doubling time, cells were grown to early log phase at 32°, and cell density was determined at regular intervals with a microscope and hemocytometer.

### Immunofluorescence microscopy

**Figure 2:**
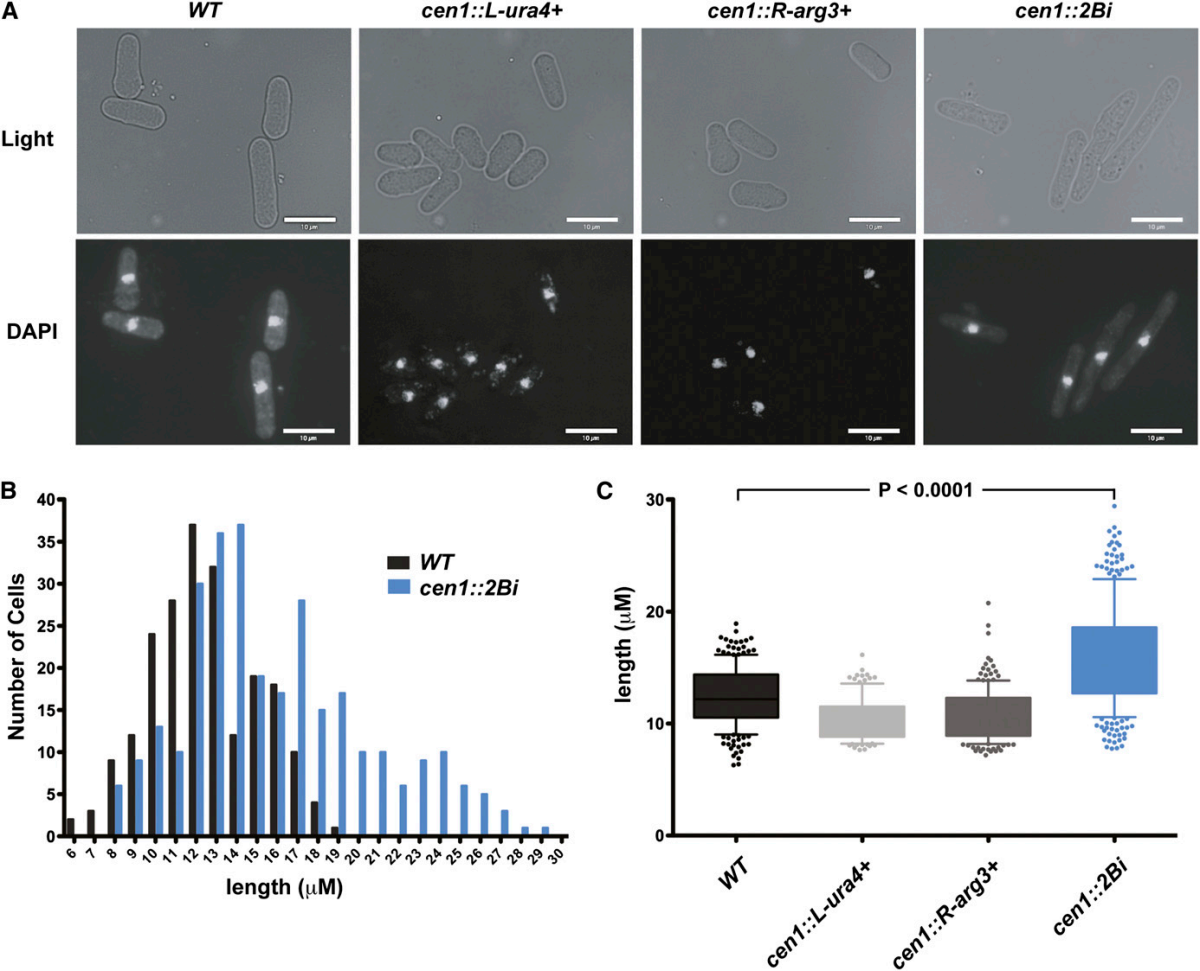
The *cen1*::*2Bi* mutant displays abnormal morphology. (A) Light and fluorescence microscopy images of the indicated ethanol-fixed strains with 4′6-diamidino-2-phenylindole (DAPI) staining of the DNA. (B) Frequency distribution of wild-type (WT) and *cen1*::*2Bi* cell length. WT, N = 211; *cen1*::*2Bi*, N = 298. (C) Cell length (μM) of indicated strains. Whiskers represent the 10th and 90th percentiles and outliers are indicated as solid circles. *cen1*::*L-ura4^+^*, N = 115; *cen1*::*R-arg3^+^*, N = 182. Graphs in (B) and (C) were generated from the same WT and *cen1*::*2Bi* data sets.

Cells in Figure 2 and Figure S1 and Figure S2 were grown to log phase at 32° in PMG media under the appropriate selection conditions. Approximately 5 × 10^6^ cells were pelleted at 3000×g and fixed in 1ml of 70% ice-cold ethanol while vortexing. An aliquot of cells was pelleted again and resuspended in 1 mL of 50mM NaCitrate and incubated at room temperature for 20 min. After precipitation and resuspension in water, cells were spread on a poly-L-lysine−coated coverslip, inverted onto 8 μL of Vectashield containing 4′6-diamidino-2-phenylindole (DAPI), and the coverslip was sealed with nail polish. Cells were examined by fluorescence microscopy using a Zeiss Axiovert 200MT microscope. Images were captured with Openlab 5.1.0 and processed with Photoshop software. P values for comparing cell lengths were generated using a two-tailed unpaired *t*-test (GraphPad Prism 5.0 software).

**Figure 3:**
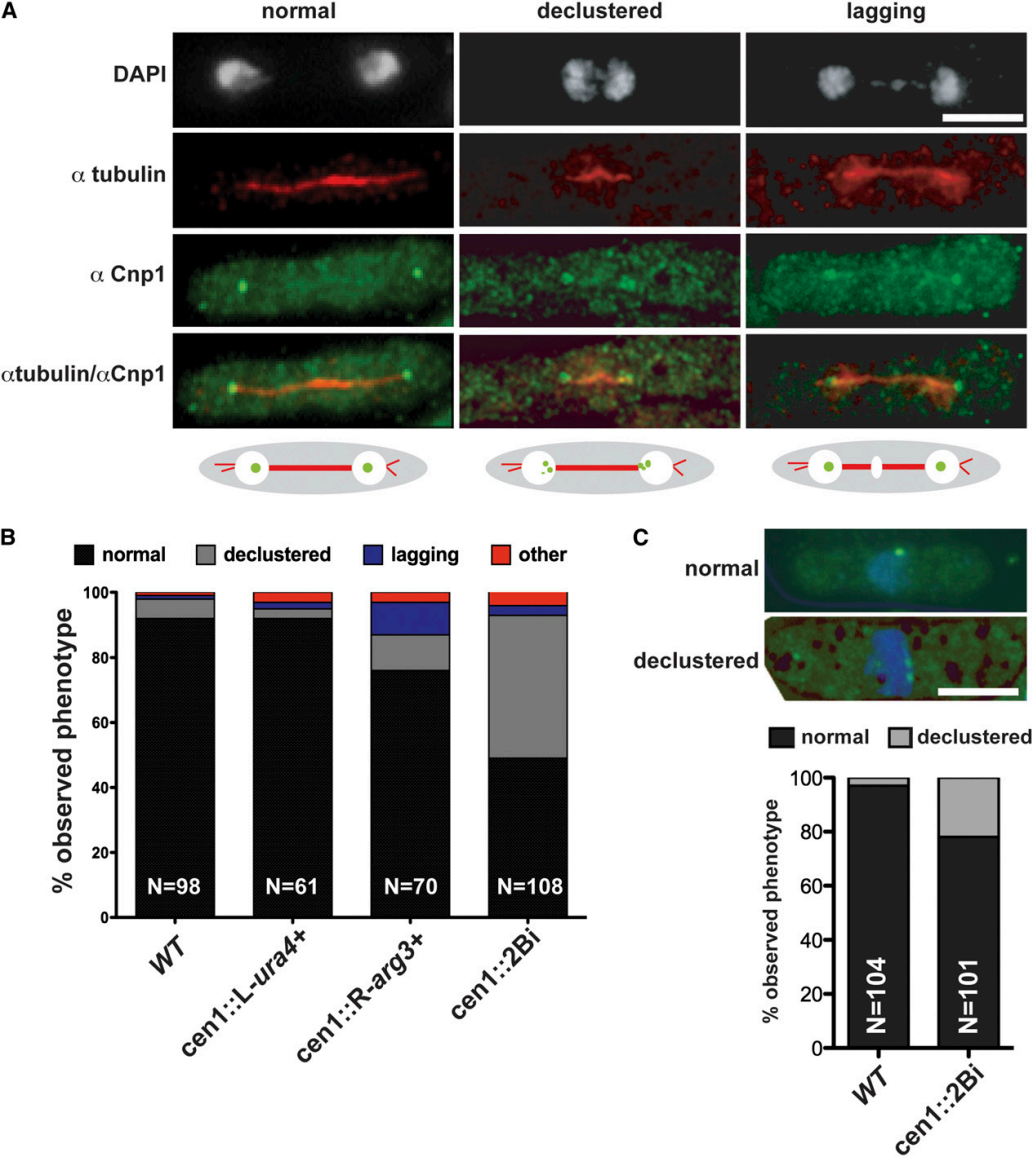
The *cen1*::*2Bi* mutant is defective in chromosome segregation and centromere clustering. (A) Representative fluorescence microscopy images of wild-type, declustered, and lagging chromosome mitotic phenotypes. Cells were fixed with paraformaldehyde and processed for immunofluorescence with tubulin antibodies to identify mitotic cells (α tubulin, red), nuclei (DAPI, white), and Cnp1 antisera to label centromeres (αCnp1, green). (B) Average percentage of cells with normal (wild-type), declustered, lagging, or other chromosome segregation phenotypes. (C) Representative fluorescence microscopy images of wild type and mutant interphase phenotypes. Cells were prepared as describe previously, and interphase cells were identified as those with a single nucleus (DAPI, blue) that lack a mitotic spindle. The average percentage of cells with normal (wild-type) or declustered Immunofluorescent signals is indicated. Scale bar, 5 μM.

Cells in Figure 3 were grown to log phase at 32° in PMG media under the appropriate selection conditions and fixed for 10 min with 3.7% freshly made paraformaldehyde (Sigma-Aldrich). Cells were washed and resuspended at 1 × 10^8^ cells/mL in PEMS (100 mM piperazine-N,N′-bis(2-ethanesulfonic acid) (PIPES), 1 mM MgCl_2_, 1 mM ethylenediaminetetraacetic acid, 1.2 M sorbitol) containing 1 mg/mL zymolyase 100T, followed by a 90-min incubation at 37° with shaking to form spheroplasts. Spheroplasts were washed in PEMS and incubated for 1 min at room temperature in PEMS containing 1% Triton X-100. Cells were washed and blocked in PEM plus 5% Donkey Normal Serum (Jackson ImmunoResearch), 100 mM lysine hydrochloride, and 0.1% sodium azide. Antibodies used were TAT1 (antitubulin; 1:50 a gift from K. Gull) and Cnp1 (anti-CENP-A^Cnp1^ antiserum, 1:100, a gift from R. Allshire and A. Pidoux). Alexa Fluor 488- and Cy3-conjugated antibodies (Jackson ImmunoResearch) were used at 1:300 and 1:200, respectively. Microscopy was performed as described previously.

### Chromatin immunoprecipitation (ChIP)

ChIP was performed as previously described (Pidoux *et al.* 2004). Cells were spheroplasted at 1 × 10^8^ cells/ml in PEMS + 0.4 mg/mL zymoloyase-100T for 25 min at 37° with shaking. Cells were washed twice in PEMS and frozen at −80°. We used 7 μL of Cnp1 antisera (A. Pidoux and R. Allshire, University of Edinburgh, Wellcome Trust Centre for Cell Biology), 2 μL of αH3K9me3 (Upstate 07−442) or 2 μL of αH3 C-term (ab1791; Abcam) was used in ChIPs. The starting quantities of input and immunoprecipitated fractions were determined from the appropriate standard curve by iQ5v.2.1 detection software (an average of two experimental replicates). The relative amount of enrichment was determined as followed: for the input PCR, the experimental locus value was normalized to input *act1^+^* locus value, giving the input ratio. Enrichment of the protein of interest at the experimental locus was calculated relative to the *act1^+^* value and then corrected for the ratio obtained in the input PCR. Standard deviations were calculated for at least six samples from at least two independent experiments. *P* values for comparing relative enrichment were generated with a two-tailed unpaired *t*-test (GraphPad Prism 5.0 software). No antibody controls were performed as a negative control, but data were not used to calculate the relative level of enrichment. Primers are listed in Table S2.

### Quantitative PCR (qPCR)

Real-time qPCR was performed in the presence of IQ SYBR Green Supermix (Bio-Rad) using the BioRad iCycler PCR system and the associated iQ5v.2.1 detection software. A standard curve for each primer set was generated with genomic DNA isolated from the wild-type strain (KFY2). The correlation coefficient for each standard curve was >0.99. The efficiency of PCR was between 90 and 110%.

### Reporter loss assay

Cells were grown to log phase at 32° in PMG media under the appropriate selection conditions. An aliquot of cells was precipitated, washed, and resuspended in YES media at ~3 × 10^6^ cells/mL. Cells were returned to 32° and grown with shaking for 5 hr. Cells were diluted to 3 × 10^3^ cells/mL and 100-μL aliquots plated on YES and PMG-ura-ade plates. YES plates were incubated at 32° for 3 d and PMG-ura-ade plates for 5 d before colonies were counted.

## Results

### Barrier deletion results in minichromosome mitotic instability

We previously demonstrated that deletion of tDNA^Ala^ at cen1, or impairment of its transcriptional activity, results in the spread of pericentric heterochromatin into the core domain ~600 bp beyond its normal boundary. The lack of tDNA^Ala^ barrier activity also results in reduced spore viability and meiotic chromosome segregation defects, suggesting a functional relationship among subdomain size, barrier activity, and genome stability. Although deletion of the nearby tDNA^Glu^ does not significantly alter cen1 chromatin structure, we proposed that tDNA^Ala^ and tDNA^Glu^ were functionally redundant barrier elements (Scott *et al.* 2006). Notably, strains lacking a 1.7-kb imr1 fragment (Figure 1A, purple line) containing both tRNA^Ala^ and tRNA^Glu^ were not recovered in previous studies, suggesting that this region of the centromere could be critical for viability (Scott *et al.* 2006). As an alternative approach to studying barrier activity on an endogenous centromere, we assayed the mitotic stability of a nonessential cen1 minichromosome pSp(cen1)-3C; Figure 1A; Hahnenberger *et al.* 1989, 1991 in the presence and absence of the 1.7-kb tDNA^Ala^-tDNA^Glu^ fragment. Wild-type strains lose the parent minichromosome at a frequency of 3.1 × 10^−3^ per cell division, in agreement with previous analyses (Hahnenberger *et al.* 1991). In the absence of the tDNA^Ala^-tDNA^Glu^ fragment, the frequency of pCen1-3C loss increases fivefold to 1.6 × 10^−2^ per cell division. From these studies we re-define the cen1 chromatin barriers as 1.7-kb fragments that flank the core chromatin subdomain. The cen1 barriers share 100% sequence identity and each contains a tDNA^Ala^ and tDNA^Glu^ gene (Wood *et al.* 2002). Each homologous barrier functions locally to maintain the structural independence of neighboring core and pericentric heterochromatin domains.

### A novel strain to study centromeric barrier activity

We generated a series of strains that contained nonidentical reporter gene insertions on the left and right side of endogenous cen1 (Figure 1B). All strains carried an *arg3^+^* reporter gene inserted into the cen1 barrier to the right of the core domain, between the tDNA^Ala^ and tDNA^Glu^ genes (*cen1*::*R-arg3^+^*, Figure 1B). Reporter genes inserted at this position are stably maintained in the absence of selection, subject to reversible position-effect variegation, and have no effect on centromere activity (Figure 1B) (Allshire *et al.* 1995; Scott *et al.* 2006). In the *cen1*::*R-arg3^+^* background, the *ura4^+^* gene was integrated at one of five different cen1 positions (Figure 1B, inverted red triangles). Analogous to previous results (Ekwall *et al.* 1999; Pidoux 2003) most strains bearing two reporter genes at a single centromere maintained wild-type growth and resistance to a microtubule poison, thiobendazole, TBZ (sites 2−5; Figure 1B). These data confirm that disruption of the inverted symmetry of cen1 has no effect on centromere function. In contrast, strains carrying a second insert into the cen1 barrier to the left of the core domain (*cen1*::*2Bi (1)*; Figure 1B) displayed severe growth defects, including sensitivity to thiabendazole (TBZ) and the accumulation of Phloxin B, an indication of increased cell death. We refer to this strain as *cen1*::*2Bi* (*i.e.*, **cen1 2 B**arrier **i**nsertion). In comparison with strains with control insertions into either the left (*cen1*::*L-ura4^+^*) or right (*cen1*::*R-arg3^+^*) cen1 barrier, *cen1*::*2Bi* strains display a growth defect and a doubling time of ~4 hr in minimal media (Figure 1C). Thus, we conclude that the disruption of both cen1 barriers on the endogenous *S. pombe* chromosome 1 results in slow growth, decreased viability, and defects in centromere activity.

### The cen1::2Bi mutant displays defects in chromosome segregation

We next examined the cell and nuclear morphology of wild type and mutant cells using DAPI to stain DNA. In addition to the growth defects observed above (Figure 1, B and C) the *cen1*::*2Bi* mutant exhibits a striking elongated cell morphology (Figure 2A), which is indicative of cell-cycle delay. Most elongated cells contain a single, undivided nucleus and have an average length of 15.9 ± 0.3μm, a significant increase in comparison to mononucleated *wild-type* cells (12.3 ± 0.2 μm; *P* < 0.001) and single-insertion control cells (Figure 2, B and C). Binucleated *cen1*::*2Bi* cells also showed an elongated morphology (20.9−0.6 μm) relative to *wild-type*, binucleated cells (15.9 ± 0.7 μm; Figure S1).

To further characterize centromere behavior at the cellular level, centromere position was examined in mitotic cells via a cytologic approach. At mitosis the duplicated wild-type *S. pombe* centromeres attach to the mitotic spindle, separate, and rapidly move to the spindle poles before nuclear and cellular division (Funabiki *et al.* 1993; Nabeshima *et al.* 1998). Centromeres on the separated chromatids appear clustered as a single focus from mid-anaphase until the following mitosis, and individual centromeres are rarely visible cytologically (Takahashi *et al.* 2000; Gregan *et al.* 2007). *Wild-type* and *cen1*::*2Bi* mitotic cells were identified by the presence of an elongated mitotic spindle, visualized by antitubulin immunofluorescence. Centromeres were immunolocalized with anti-CENP-A^Cnp1^ antiserum and DNA stained with DAPI (Figure 3A). As expected, 92% of wild-type cells displayed the normal chromosome segregation phenotype (N = 98; Figure 3B). The single insert control strain *cen1*::*L-ura4^+^* segregated similarly to the wild-type strain (92% normal segregation, N = 61). Strain *cen1*::*R-arg3^+^* displayed a slight increase in the fraction of cells with mitotic defects (77% normal segregation, N = 70), despite having no observable phenotype on media containing TBZ or Phloxin B (Figure 1B) and normal cell morphology (Figure 2, A and B). We are unclear what causes the modest reduction in the fidelity of chromosome segregation in *cen1*::*R-arg3^+^*, but not *cen1*::*L-ura4^+^* strains (see *Discussion*).

In contrast to the single-insert strains, a large fraction of the *cen1*::*2Bi* mutant cells displayed abnormal mitotic phenotypes. In 44% of the cells (N = 108), the normally colocalized centromeres of segregating chromatids appear diffuse at either one or both of the anaphase poles (Figure 3, A and B). We typically observed between two and four CENP-A^Cnp1^ signals associated with the ends of the mitotic spindle. Other nuclear and mitotic defects were observed in *cen1*::*2Bi* cells, often in combination with the declustered phenotype, including lagging chromosomes, unequal segregation of DNA, and decondensed DNA.

Clustering of centromeres also occurs throughout interphase in wild-type cells, although the mechanism and functional importance of this arrangement is poorly understood. Interphase cells were identified as mononucleated cells that lack a mitotic spindle, and the centromere position was visualized by anti-CENP-A^Cnp1^ antiserum. In *wild-type* and single-insert control strains, we observed 97% of the cells displayed a single, intense fluorescent signal, indicative of centromere clustering, as expected (Funabiki *et al.* 1993). In contrast, 22% of *cen1*::*2Bi* cells displayed dispersed punctate CENP-A^,Cnp1^ fluorescence signals, indicative of a mild defect in centromere clustering (Figure 3C). Notably, despite the defect in centromere position in *cen1*::*2Bi* interphase cells, the CENP-A^Cnp1^ signals remained in close proximity to one another. We conclude that the simultaneous interruption of both cen1 chromatin barriers is incompatible with normal centromere activity.

### Barrier activity is maintained in cen1::2Bi mutants

**Figure 4:**
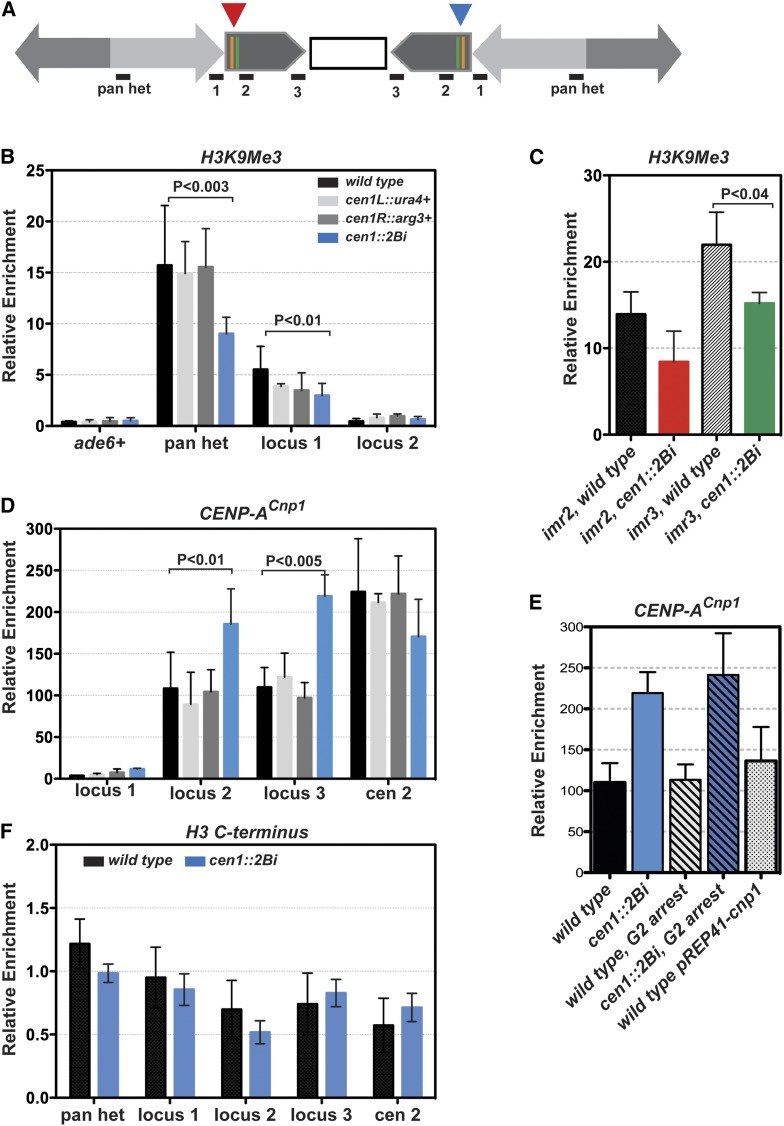
Lack of barrier activity affects the structure of both core and pericentric heterochromatin domains. (A) The structure of *cen1*::*2Bi* centromere 1 and amplicon loci. (B) ChIP-qPCR average relative enrichment of H3K9Me in wild-type (black) cen1::L-*ura4^+^* (light gray), cen1::R-*arg3^+^* (dark gray), and cen1::2Bi (blue) cells. (C) ChIP-qPCR average relative enrichment of H3K9Me in wild type (black and patterned) and cen1::2Bi cells (red, green) at imr2 and imr3 pericentric heterochromatin loci. (D) ChIP-qPCR average relative enrichment of CENP-A^Cnp1^ as in B. (E) ChIP-qPCR average relative enrichment of CENP-A^Cnp1^ at locus 3 in asynchronous (solid bars), G2 arrested (striped bars) and CENP-A^Cnp1^ overexpressing (dotted bar) yeast strains. (F) ChIP-qPCR average relative enrichment of histone H3 C-terminus in wild type (black) and *cen1*::*2Bi* cells (blue). Each ChIP-qPCR was performed in duplicate in at least three independent experiments. Relative enrichment at each locus was calculated from standard curve data (see Materials and Methods) and normalized to input and *act1^+^*. Error bars represent the standard deviation among the six or more data points.

The insertion of a reporter gene into one or the other cen1 chromatin barrier has no effect on centromere function (Allshire *et al.* 1995; Scott *et al.* 2006) (Figure 1). In contrast, the partial deletion of one heterochromatin barrier at endogenous cen1 results in the spread of pericentric heterochromatin beyond its normal boundary and defects in meiotic chromosome segregation (Scott *et al.* 2006, 2007). Thus, we considered the possibility that insertional mutagenesis into *both* chromatin barriers at cen1 in *cen1*::*2Bi* cells might neutralize barrier activity, resulting in the encroachment of pericentric heterochromatin into the core domain and the observed mitotic defects. To test this hypothesis, we used ChIP with antibodies specific for the heterochromatin-specific modification, H3K9me3. Immunoprecipitated DNA was quantified by real-time, qPCR. In contrast to the hypothesis, the boundaries between core chromatin and heterochromatin are maintained at cen1 in *cen1*::*2Bi* cells; H3K9me3 enrichment is significantly above background at locus 1 and depleted at locus 2 in all strains tested (Figure 4, A and B). These data suggest that barrier activity, defined as the ability to block the spread of heterochromatin (Sun and Elgin 1999), remains uncompromised in *cen1*::*2Bi* cells. Barrier function was independently confirmed by analysis of the steady-state reporter gene transcript levels in *cen1*::*2Bi* strains. We observed no significant difference in *ura4^+^* or *arg3^+^* gene expression between *cen1*::*2Bi* cells when compared to the corresponding single reporter gene insert control (Figure S2). Thus, in contrast to partial barrier deletion of a single heterochromatin barrier (Scott *et al.* 2006), the local heterochromatin blocking activity of both cen1 chromatin barriers is maintained in *cen1*::*2Bi* strains.

### Aberrant centromere chromatin integrity in *cen1*::*2Bi* mutant

Although the boundaries of H3K9me3 chromatin is maintained in *cen1*::*2Bi* cells, we observed a modest, but significant, reduction in the density of H3K9me3 enrichment at locus 1 (Figure 4B: wild-type, 5.5 ± 0.8; *cen1*::*2Bi*, 3.0 ± 0.4; *P* < 0.01) and a strong reduction at a heterochromatin repeat element, common to all three centromeres (pan het; wild type, 17.1 ± 2.0; *cen1*::*2Bi*, 9.0 ± 0.7, *P* < 0.003). The panhet primers used in this assay are predicted to amplify nine different genomic loci, only two of which are present on cen1. Thus, these results strongly suggest that heterochromatin domain integrity is compromised at all centromeres in *cen1*::*2Bi* strains. To test this, we designed primers to specifically query heterochromatic loci at centromere 2 (cen2) and centeromere 3 (cen3). We observed a modest reduction in the density of H3K9me3 at both cen2 and cen3 loci in the cen1::2Bi mutant. (Figure 4C). Thus, the site-specific insertions in the cen1::2Bi mutant alter the integrity of all three centromeres.

We next examined the distribution of CENP-A^Cnp1^ in the *cen1*::*2Bi* mutant by ChIP. CENP-A^Cnp1^ is absent from the heterochromatin domain in all strains tested (Figure 4C, site 1), further validating the conclusion that the boundary between centromeric subdomains is intact in *cen1*::*2Bi* cells. Depletion of CENP-A^Cnp1^ in wild-type cells results in an increase in chromosome mis-segregation events (Takahashi *et al.* 2005); thus, we hypothesized that the *cen1*::*2Bi* phenotypes could result from an insufficient amount of CENP-A^Cnp1^ at cen1. Remarkably, we found a consistent twofold increase in CENP-A^Cnp1^ enrichment at the cen1 core domain in *cen1*::*2Bi* strains, as compared to *wild-type* centromeres and single insertion strains (Figure 4D, loci 2 and 3). The striking structural changes in the cen1 core are unlikely due to an indirect effect, such as a second site mutation or a change in the transcription or stability of unincorporated CENP-A^Cnp1^ because there is no significant difference in the relative enrichment of CENP-A^Cnp1^ at cen2 among all strains tested (Figure 4D, cen2).

An increase in the relative amount of CENP-A^Cnp1^ at cen1 in *cen1*::*2Bi* may be due to an increase in the average number of nucleosomes at cen1 or, alternatively, to an increase in the number of CENP-A^Cnp1^−containing nucleosomes at cen1. To distinguish between these possibilities, we performed ChIP analysis on sheared chromatin with an average size of 700 bp using an antibody that recognizes the C-terminus of both histone H3 and CENP-A^Cnp1^. The antibody does not distinguish among histone H3 and its variants (Castillo *et al.* 2007); it can instead be used to determine the relative number of nucleosomes present at a specific locus. At all loci tested, we observe no significant change in the average number of nucleosomes between wild type and *cen1*::*2Bi* centromeres (Figure 4F). These data suggest that, although the average number of cen1 core domain nucleosomes is similar between *wild-type* and *cen1*::*2Bi* strains, there is an increase in the average number of nucleosomes that contain CENP-A^Cnp1^ when cen1 barriers are modified by insertional mutagenesis.

Endogenous *S. pombe* centromeres are not saturated with CENP-A^Cnp1^ (Castillo *et al.* 2007). Those data, combined with the observation that *cen1*::*2Bi* cells have an extended doubling time (Figure 1C) and an elongated phenotype (Figure 2), suggest that the increase in CENP-A^Cnp1^ enrichment could be a consequence of a prolonged CENP-A^Cnp1^ loading period in *cen1*::*2Bi* cells. In contrast to multicellular eukaryotes, *S. pombe* CENP-A^Cnp1^ is deposited in G2, with a maximum amount of CENP-A^Cnp1^ detected in late G2 (Lando *et al.* 2012). Thus, if barrier insertions result in extended CENP-A^Cnp1^ loading period, then we predict that *wild-type* cells arrested in G2 would have a level of CENP-A^Cnp1^ enrichment comparable with *cen1*::*2Bi* cells. To test this hypothesis, both *wild-type* and *cen1*::*2Bi* cells were arrested as elongated cells in G2 with a temperature-sensitive *cdc25-2* mutant, and CENP-A^Cnp1^ association was analyzed by ChIP. We observed that arrested *cen1*::*2Bi* cells remained ~2X enriched compared with control, *wild-type* cells (Figure 4E). We also analyzed CENP-A^Cnp1^ enrichment cells that overexpress CENP-A^Cnp1^ and observed a modest, but insignificant increase in CENP-A^Cnp1^ enrichment at the *wild-type* centromere. Thus, we conclude that the twofold increase in CENP-A^Cnp1^ at cen1 in *cen1*::*2Bi* cells is a consequence of neither an extended CENP-A^Cnp1^ loading period nor an increase in average cellular amount of CENP-A^Cnp1^.

### Centromere activity is restored after reporter gene loss

In contrast to the slow growth defects observed in minimal media, we noted that *cen1*::*2Bi* cells grow nearly as well as *wild-type* cells in rich media (Figure S3). Phenotypic analysis revealed that cultures grown in rich media were no longer viable on media lacking uracil and arginine, suggesting that one or both of the barrier-embedded reporter genes had been mutated or eliminated from the *cen1*::*2Bi* genome. To distinguish between these possibilities, genomic DNA was amplified with primers that flank the cen1 barriers. Amplicons consistent with the elimination of the barrier-embedded reporter gene were detected in 100% of colonies amplified (N = 192), and sequence analysis confirmed complete excision of the reporter gene and restoration of the wild type sequence (Figure S3). Thus, the barrier-embedded reporter genes present in *cen1*::*2Bi* mutant strains are unstable.

**Figure 5:**
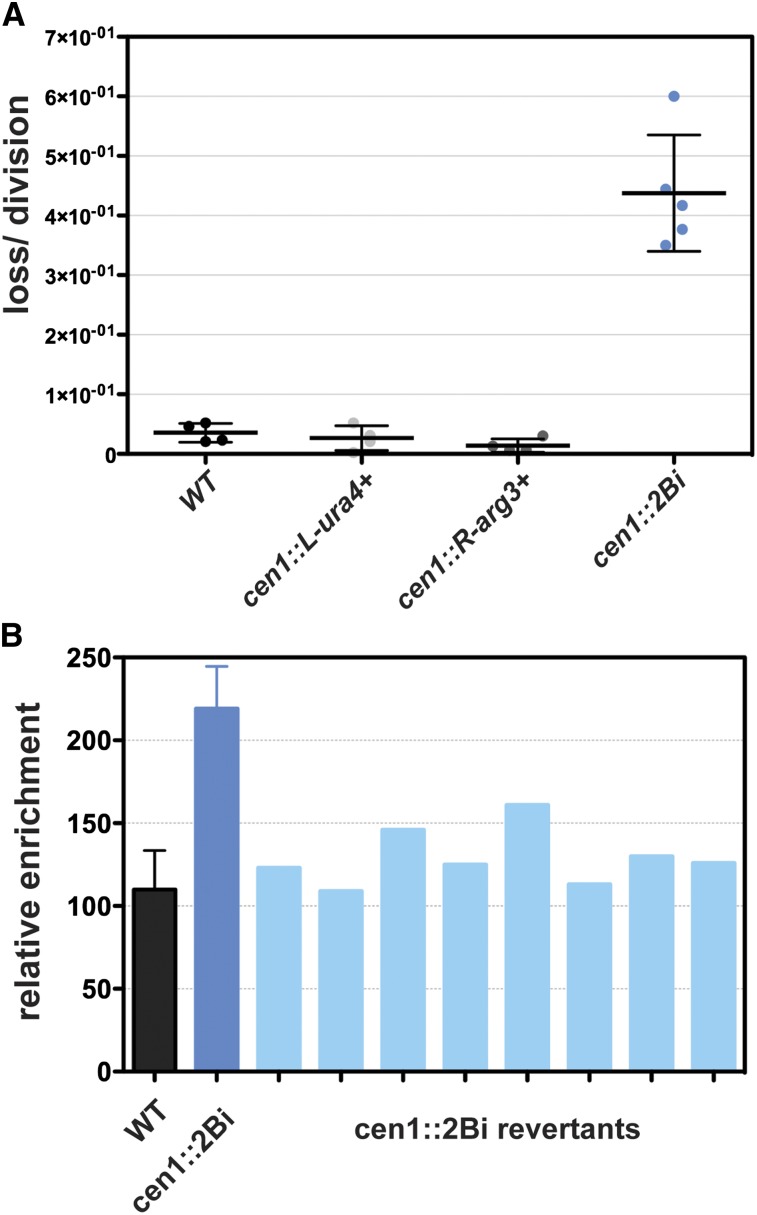
The *cen1*::*2Bi* embedded reporter genes are unstable. (A) Frequency of *ura4^+^ arg3^+^* auxotrophy in indicated strains. The dotted line for each strain represents the mean and the error bars indicate the standard deviation. Data points are independent biological replicates. (B) ChIP-qPCR average relative enrichment of CENP-A^Cnp1^ in *wild type* (WT), *cen1*::*2Bi*, and *cen1*::*2Bi* revertants at locus 3, illustrated in Figure 4A. Wild-type and *cen1*::*2Bi* data are identical to data in Figure 4C. Each light blue bar represents an independently isolated revertant strain, for which a single ChIP-quantitative polymerase chain reaction experiment was performed.

To estimate the frequency of reporter gene loss, wild-type, *cen1*::*L-ura4^+^*, *cen1*::*R-arg3^+^*, and *cen1*::*2Bi* strains were grown to logarithmic phase under selective conditions, washed, and grown in rich media for 1−2 doublings (5 hr). Cells were then plated at low density on rich media and selective media to estimate the percentage of cells that maintained one (*cen1*::*L-ura4^+^*, *cen1*::*R-arg3^+^*) or both (*cen1*::*2Bi*) barrier-embedded reporter genes. We determined that control and single insert strains became auxotrophic for *ura4^+^* and/or *arg3^+^* at a low frequency whereas the frequency is increased ∼15 fold in *cen1*::*2Bi* mutants (Figure 5A).

The level of CENP-A^Cnp1^ enrichment at cen1 was examined in several independent *cen1*::*2Bi* revertant strains by ChIP. The data demonstrate that each revertant strain possesses wild-type levels of CENP-A^Cnp1^ (Figure 5B). Thus, the growth defects and changes in chromatin structure observed in cen1::2Bi cells are completely reversible.

## Discussion

### Barriers are required for accurate chromosome segregation in fission yeast

Chromatin insulators and barriers have well-defined roles in regulating gene expression and the protection against position-effect variegation (Ghirlando *et al.* 2012; Yang and Corces 2012). Here, we demonstrate a requirement for centromere-specific chromatin barriers in chromosome segregation and the maintenance of genome stability. Although it remains a formal possibility that an inherent feature of one or both of the embedded reporter genes results in the severe phenotypes observed in *cen1*::*2Bi* cells, we do not favor this hypothesis because the inserted reporter genes have similar features. The total insertion size on the left and right of the cen1 core domain was kept constant (1.7 kb) and the *ura4^+^* and *arg3^+^* promoters possess comparable promoter activity (Lackner *et al.* 2007).

Previous studies in both multicellular eukaryotes and *S. pombe* have demonstrated a role for insulators/ barriers in higher-order genome organization (Tolhuis *et al.* 2002; Blanton *et al.* 2003; Byrd and Corces 2003; Noma *et al.* 2006), suggesting that centromeric barriers could similarly contribute to higher-order centromere organization. Recent studies implicate topoisomerases and helicases as important regulators of core chromatin integrity in *S. pombe*, consistent with the idea that the higher-order structures provide a chromatin context that is ensures accurate chromosome segregation (Norman-Axelsson *et al.* 2013). Thus, we speculate that centromeric chromatin barriers play an important role in locally organizing centromere architecture, thus facilitating the appropriate deposition and/or removal of CENP-A^Cnp1^, which in turn ensures proper spindle attachment and chromosome segregation.

### Centromeric chromatin domains are interdependent in *S. pombe*

Classic and reverse genetic approaches, performed in a variety of organisms, have produced an inventory of dozens of centromere proteins that contribute to the establishment and maintenance of either the core/kinetochore or pericentric heterochromatin subdomain-but not both (Pidoux and Allshire 2004; Verdaasdonk and Bloom 2011). Thus, it is striking that the modification of homologous chromatin barriers can affect the integrity of both centromeric chromatin subdomains, without disturbing the boundary between them. This suggests that centromeric subdomain maintenance is interdependent, and coordinated by both genomic and epigenetic processes. Our data also suggest that the integrity of the pericentric heterochromatin domains can be compromised on unlinked centromeres (imr2, imr3; Figure 4, B and C). This raises the intriguing possibility that the disruption of barrier activity on a single centromere can influence the segregation of other chromosomes in the same nucleus, in *trans*. Together, these data extend the conclusion that the proper organization, integrity and balance of centromeric chromatin subdomains is critical to centromere identity and genome stability (Nakano *et al.* 2008; Bergmann *et al.* 2011; Sullivan *et al.* 2011; Slee *et al.* 2012).

### Mitotic recombination at centromeres

Somatic recombination events have been detected at centromeres of many organisms (Liebman *et al.* 1988; Jaco *et al.* 2008; Shi *et al.* 2010) likely due to the presence of repetitive sequences and their potential instability (Talbert and Henikoff 2010). Mitotic recombination can also occur at fission yeast centromeres, although these events have been difficult to study experimentally due to shared sequence homology among the three centromeres. By taking advantage of nonessential minichromosome assays and engineered experimental loci, limited studies have demonstrated that recombination events at the centromere are typically suppressed by the assembly of pericentric heterochromatin and the presence of replication fork stability proteins (Li *et al.* 2013). The Rad51 homologous recombination protein also limits isochromosome formation and other recombination based chromosomal rearrangements at the centromere (Nakamura *et al.* 2008).

On the basis of the perfect sequence identity of the inverted repeats within the *S. pombe* centromeres, it has been suggested that a recombination-based mechanism is involved in maintaining higher order centromere structure (Takahashi *et al.* 1992; McFarlane and Humphrey 2010). The observed rapid and efficient excision of exogenous DNA from *cen1*::*2Bi* strains is consistent with this model, although it remains unclear whether these events normally occur at *wild-type* centromeres. Recombination and repair could result from collisions among the DNA metabolism machinery including RNA polymerase II, RNA polymerase III and replication fork complexes (Li *et al.* 2013). Alternatively, an increase in the distance or a change in the AT content between tDNA^Ala^ and tDNA^Glu^ at each barrier could cause topological constraint at the centromere, exposing single-stranded DNA that is more susceptible to breakage. These and other models wait further experimental testing.

## Supplementary Material

Supporting Information
