# Supplementary material for: Centromeric Barrier Disruption Leads to Mitotic Defects in Schizosaccharomyces pombe
Source: G3 (Bethesda). 2014 Feb 13;4(4):633–42. doi: 10.1534/g3.114.010397 (PMC4059236; doi:10.1534/g3.114.010397)
Supplement: Supporting Information [file supp_4_4_633__index.html]

Centromeric Barrier Disruption Leads to Mitotic Defects in Schizosaccharomyces pombe — Supporting Information 

# Centromeric Barrier Disruption Leads to Mitotic Defects in *Schizosaccharomyces pombe*

## Supporting Information for Gaither *et al.*, 2014

**Files in this Data Supplement:**

- Supporting Information - Figures S1-S3 and Tables S1-S2 (PDF, 405 KB)
- Figure S1 - The *cen1::2Bi* mutant displays abnormally long morphology. (PDF, 142 KB)
- Figure S2 - Barriers are intact in the *cen1::2Bi* mutant. (PDF, 144 KB)
- Figure S3 - The *cen1::2Bi* revertant strains have wild-type growth kinetics and sequence. (PDF, 598 KB)
- Table S1 - List of yeast strains used in this study. (PDF, 114 KB)
- Table S2 - List of primers used in this study. (PDF, 114 KB)
