# Supplementary material for: Centromeric Barrier Disruption Leads to Mitotic Defects in Schizosaccharomyces pombe
Source: G3 (Bethesda). 2014 Feb 13;4(4):633–42. doi: 10.1534/g3.114.010397 (PMC4059236; doi:10.1534/g3.114.010397)
Supplement: Supporting Information [file supp_g3.114.010397_010397SI.pdf]

G3 · Genes | Genomes | Genetics

Centromeric barrier disruption leads to mitotic defects in *Schizosaccharomyces pombe*

Terilyn L. Gaither*, Stephanie L. Merrett[1]*, Matthew J. Pun*, and Kristin C. Scott*§

*Institute for Genome Sciences and Policy
Duke University
DUMC 3382
Durham, NC 27708

§ Department of Molecular Genetics and Microbiology
Duke University Medical Center
Durham NC 27710

[1]Present Address:
Max Planck Institute of Molecular Cell Biology and Genetics
Pfotenhauerstrasse 108
01307 Dresden
Germany

Corresponding Author:
Kristin C. Scott
Institute for Genome Sciences and Policy
Duke University
101 Science Drive Box 3382
Durham NC 27708
(919) 684-7938
kristin.scott@duke.edu

DOI: 10.1534/g3.114.010397

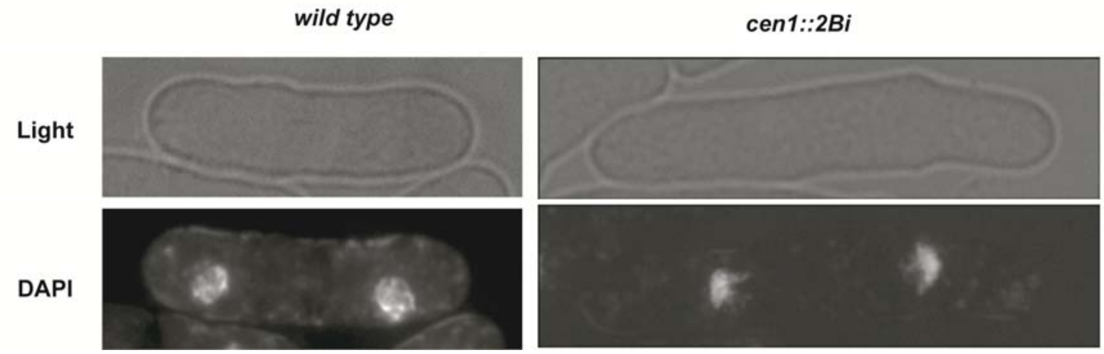

Figure S1 The *cen1::2Bi* mutant displays abnormally long morphology. Light and fluorescence microscopy images of binuclear cells of the indicated ethanol-fixed strains with DAPI staining of the DNA.

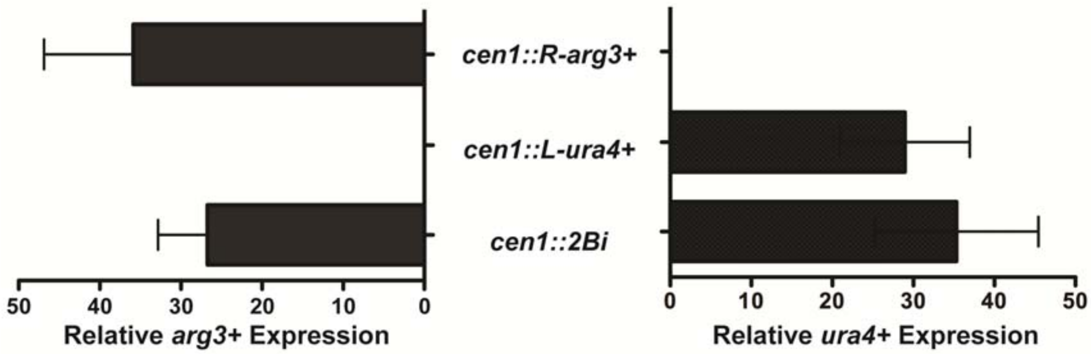

Figure S2 Barriers are intact in the *cen1::2Bi* mutant. Relative *ura4*+ and *arg3*+ gene expression in *cen1::L-ura4+, cen1::R-arg3+* and *cen1::2Bi* strains. Reporter gene expression was assayed by quantitative RT-PCR and values are reported as expression relative to an internal control, *act1*+.

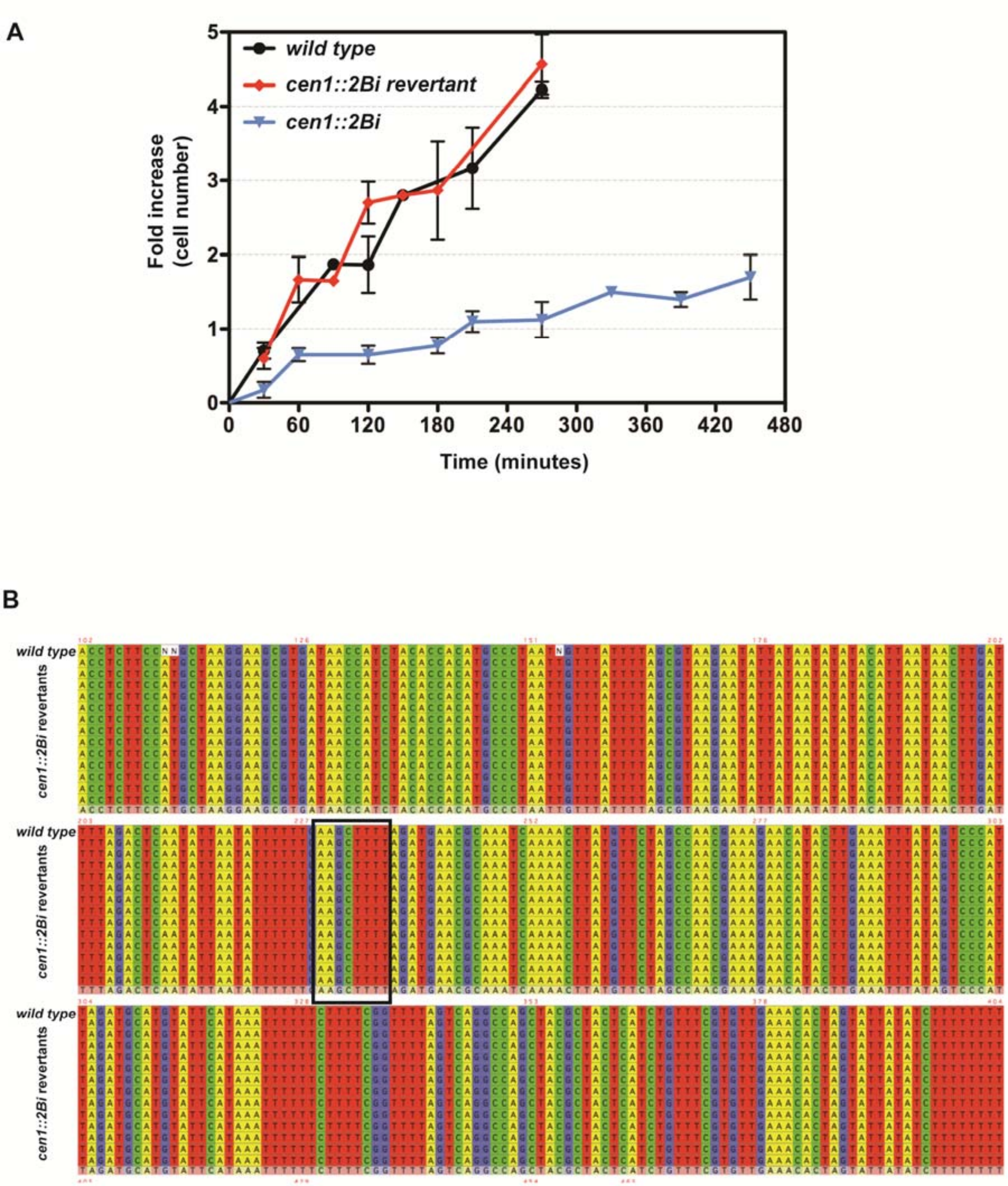

Figure S3 The *cen1::2Bi* revertant strains have wild-type growth kinetics and sequence. (A) Growth kinetics of cen1::2Bi revertant strains in non-selective media at 32°C. For comparison, the kinetics of WT and cen1::2Bi strains in selective media at 32°C are also displayed. These data are identical to those in Figure 1C. (B) Partial sequence analysis of cen1::2Bi revertant strains. The site of reporter gene insertion (Hind III site) is boxed.

Quantitative RT-PCR

Total nucleic acid was isolated from logarithmically growing cells in YES or PMG –ura-arg media at 32°C and was then subjected to DNAase treatment and RT-PCR using oligodT as a primer (Scott, *et al*., 2006). Expression was analyzed by quantitative PCR using SYBR Green on a BioRad iCycler using primers specific to the integrated copy of *ura4*$^{+}$ or *arg3*$^{+}$ and normalized to expression from the act1^{+} locus.

Table S1 List of yeast strains used in this study.

	Genotype
KFY503	*h^{90} pCen1-3C (ura3^{+}) ade6-210 leu1-32 his3D arg3D4 ura4 293*
KFY1174	*h^{90} pCen1-3C (ura3^{+})imr(ΔalaΔglu)::ade6^{+} ade6DN/N arg3D4 his3D leu1032 ura4 293*
KFY1175	*h^{90} pCen1-3C (ura3^{+})imr(ΔalaΔglu)::ade6^{+} ade6DN/N arg3D4 his3D leu1032 ura4 293*
KFY556	*h+ imr1L(Hind III)::ura4^{+} leu1-32 ade6-210 ura4D18 arg3D4 his3D*
KFY557	*h- imr1L(Hind III)::ura4^{+} leu1-32 ade6-210 ura4D18 arg3D4 his3D*
KFY1568	*h+ imr1R (HindIII)::arg3^{+} ura4D18 his3D arg4D4 leu1-32 ade6DN/N*
KFY1569	*h- imr1R (HindIII)::arg3^{+} ura4D18 his3D arg4D4 leu1-32 ade6DN/N*
KFY1597	*h- imr1R (HindIII)::arg3^{+} imr1L (HindIII)::ura4 ura4D18 his3D arg3D4 leu1-32 ade6-210*
KFY1598	*h^{90} imr1R (HindIII)::arg3^{+} imr1L (HindIII)::ura4^{+} ura4D18 his3D arg3D4 leu1-32 ade6-210*
KFY1629	*h- imr1R (HindIII)::arg3^{+} imr1L (HindIII)::ura4^{+} ura4D18 his3D arg3D4 leu1-32 ade6-210*
KFY1703	*h^{+} cdc25-22 leu1-32 ura4D18 arg3D4 his3D*
KFY1871	*h? imr1R (HindIII)::arg3^{+} imr1L (HindIII)::ura4^{+} cdc25-22 ura4D18 his3D arg3D4 leu1-32 ade6-210*
KFY1912	*h- ars1(MluI)::pREP41XCnp1(Leu2+) leu1-32*

Table S2 List of primers used in this study.

Primer Name	Sequence	Use
47332339	CGGGATCCGGTCGAAAAAAATTCATCCC	amplify *arg3*$^{+}$
47332340	CGGGATCCAAATTGATCCATCCCCTT	amplify *arg3*$^{+}$
5055	GCAAACACATTAATTCTCATGAATTTAGAGATTT CCATTAATAACTTGATTTTAGACTCAATATTAATATTT	Δala
5056	AAATATTAATATTGAGTCTAAAACAAGTTATTAATG GAAATCTCTAAATTCATGAGAATTAATGTGTTTGC	Δala
5057	AAAAAAAAAAAAAAAAGAGATAATTTTTTTATAAGC TACTTTTTATTTTGAAATTAA	Δglu
5058	TTAATTTCAAAATAAAAAGTAGCTTATAAAAAAAT TATCTCTTTTTTTTTTTTTTTT	Δglu
6705	CGCGGATCCATTCGTTFTACCAACTGCT	pcr check
6708	CGCGGATCCTCGCAGCCTTTCAATAACT	pcr check
7453	AATCCAACCGTGAGAAGATGA	Actin RT
7454	ACGACCAGAGGCATACAAAGA	Actin RT
BW84F	CGGCATCGCTTGTACTTTTT	panhet RT
BW84R	GACGGAACCAAATGATGTGA	panhet RT
5373	AAATCACCGGAGCAATGTTT	Site1 RT
5374	AAACACCATGGTTTGTTTGTTA	Site1 RT
5375	TCATTCGTTGTACCAACTGCT	Site2 RT
5376	TGTGTTTGCCATCTTACAATTCA	Site2 RT
201301	TGCGGTTGAGTGTAGGAAAA	Site3 RT
201302	CTGATAGCACATAAACTTTATCATCA	Site3 RT
201307	GCCAGACTTTCTTAGGATATGAATT	cen2het_imr2F
201308	CATCAGTTCGAAATCATTCTACTTG	cen2het_imr2R
201309	TTGTTGCCGCACTTGATG	cen3het_imr3F
201310	TCATGCATACATTACCGATCTACC	cen3het_imr3R
