# Supplementary material for: Centromeric Barrier Disruption Leads to Mitotic Defects in Schizosaccharomyces pombe
Source: G3 (Bethesda). 2014 Feb 13;4(4):633–42. doi: 10.1534/g3.114.010397 (PMC4059236; doi:10.1534/g3.114.010397)
Supplement: Supporting Information [file supp_g3.114.010397_FigureS1.pdf]

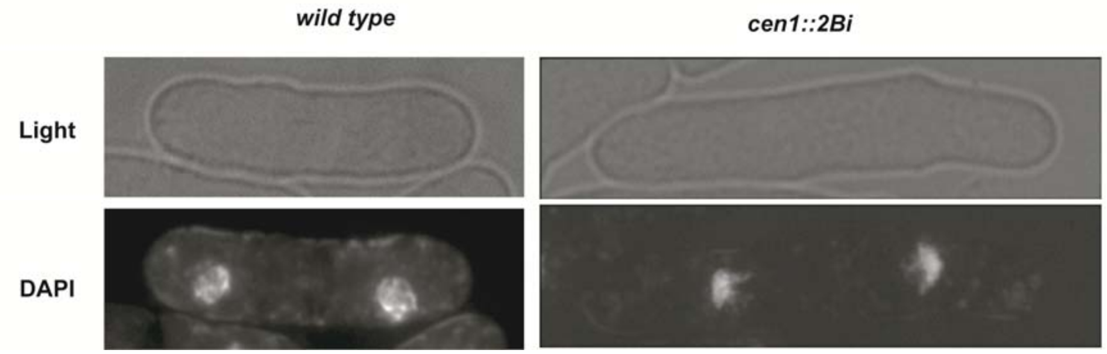

Figure S1 The *cen1::2Bi* mutant displays abnormally long morphology. Light and fluorescence microscopy images of binuclear cells of the indicated ethanol-fixed strains with DAPI staining of the DNA.
