# Supplementary material for: Centromeric Barrier Disruption Leads to Mitotic Defects in Schizosaccharomyces pombe
Source: G3 (Bethesda). 2014 Feb 13;4(4):633–42. doi: 10.1534/g3.114.010397 (PMC4059236; doi:10.1534/g3.114.010397)
Supplement: Supporting Information [file supp_g3.114.010397_FigureS2.pdf]

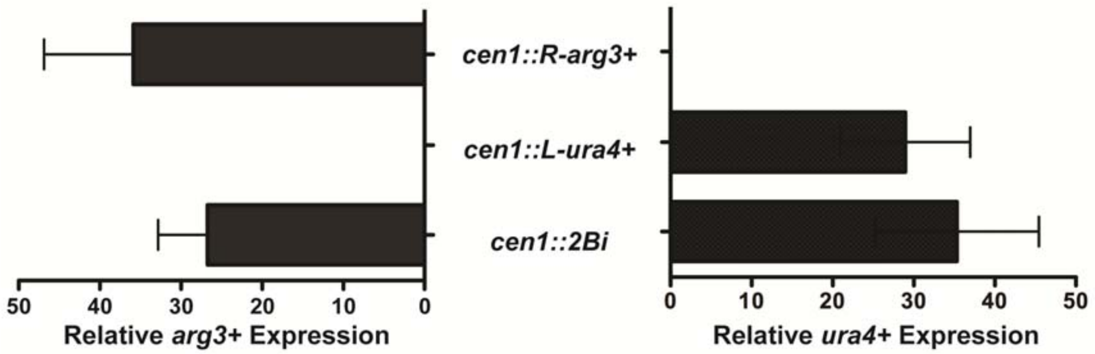

Figure S2 Barriers are intact in the *cen1::2Bi* mutant. Relative *ura4*$^{+}$ and *arg3*$^{+}$ gene expression in *cen1::L-ura4+, cen1::R-arg3+* and *cen1::2Bi* strains. Reporter gene expression was assayed by quantitative RT-PCR and values are reported as expression relative to an internal control, *act1*$^{+}$.
