# Supplementary material for: Centromeric Barrier Disruption Leads to Mitotic Defects in Schizosaccharomyces pombe
Source: G3 (Bethesda). 2014 Feb 13;4(4):633–42. doi: 10.1534/g3.114.010397 (PMC4059236; doi:10.1534/g3.114.010397)
Supplement: Supporting Information [file supp_g3.114.010397_FigureS3.pdf]

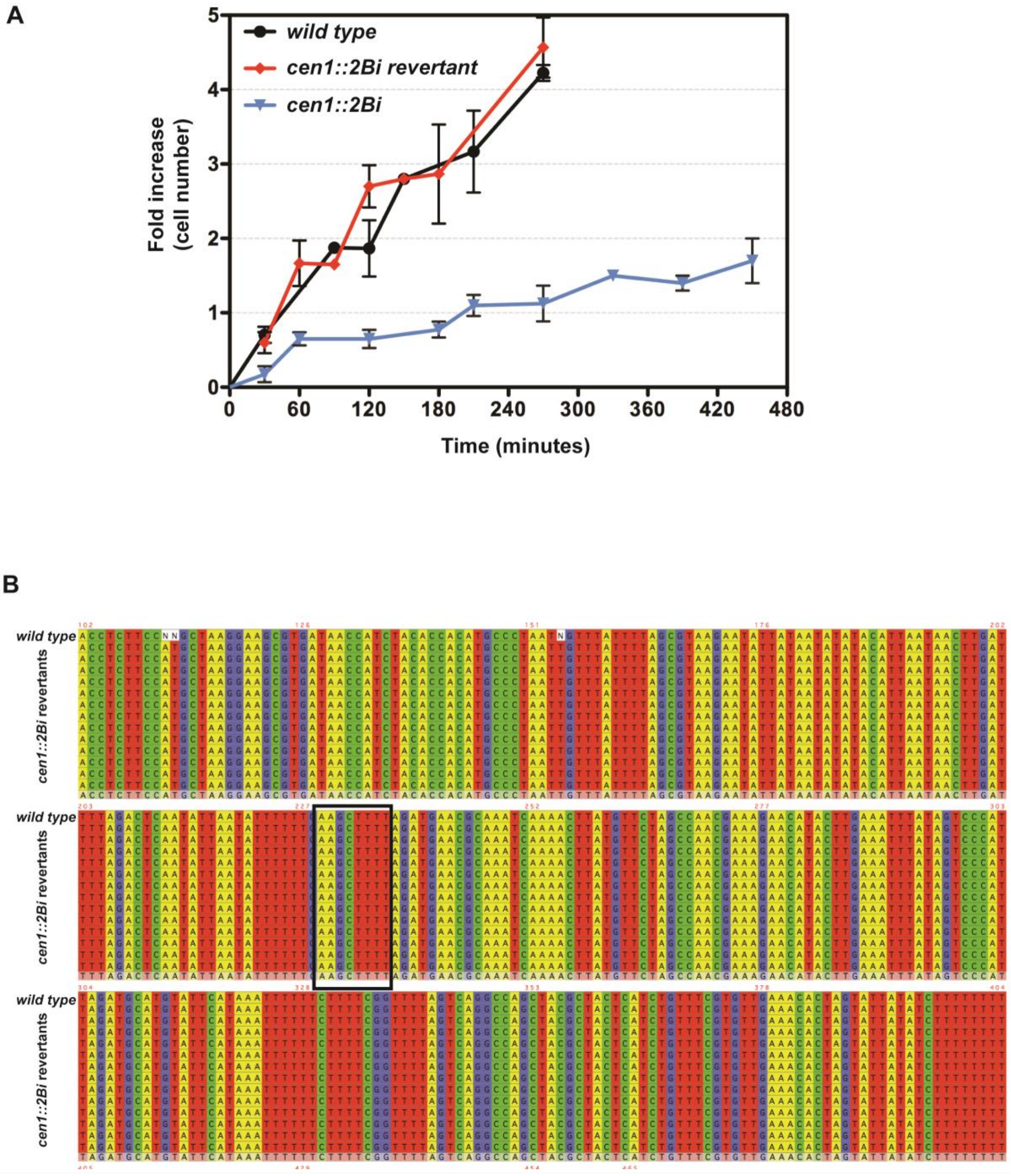

Figure S3 The *cen1::2Bi* revertant strains have wild-type growth kinetics and sequence. (A) Growth kinetics of cen1::2Bi revertant strains in non-selective media at 32°C. For comparison, the kinetics of WT and cen1::2Bi strains in selective media at 32°C are also displayed. These data are identical to those in Figure 1C. (B) Partial sequence analysis of cen1::2Bi revertant strains. The site of reporter gene insertion (Hind III site) is boxed.

Quantitative RT-PCR

Total nucleic acid was isolated from logarithmically growing cells in YES or PMG –ura-arg media at 32°C and was then subjected to DNAase treatment and RT-PCR using oligodT as a primer (Scott, *et al*., 2006). Expression was analyzed by quantitative PCR using SYBR Green on a BioRad iCycler using primers specific to the integrated copy of *ura4*$^{+}$ or *arg3*$^{+}$ and normalized to expression from the act1^{+} locus.
