# Supplementary material for: Centromeric Barrier Disruption Leads to Mitotic Defects in Schizosaccharomyces pombe
Source: G3 (Bethesda). 2014 Feb 13;4(4):633–42. doi: 10.1534/g3.114.010397 (PMC4059236; doi:10.1534/g3.114.010397)
Supplement: Supporting Information [file supp_g3.114.010397_TableS1.pdf]

Table S1 List of yeast strains used in this study.

	Genotype
KFY503	*h^{90} pCen1-3C (ura3^{+}) ade6-210 leu1-32 his3D arg3D4 ura4 293*
KFY1174	*h^{90} pCen1-3C (ura3^{+})imr(ΔalaΔglu)::ade6^{+} ade6DN/N arg3D4 his3D leu1032 ura4 293*
KFY1175	*h^{90} pCen1-3C (ura3^{+})imr(ΔalaΔglu)::ade6^{+} ade6DN/N arg3D4 his3D leu1032 ura4 293*
KFY556	*h+ imr1L(Hind III)::ura4^{+} leu1-32 ade6-210 ura4D18 arg3D4 his3D*
KFY557	*h- imr1L(Hind III)::ura4^{+} leu1-32 ade6-210 ura4D18 arg3D4 his3D*
KFY1568	*h+ imr1R (HindIII)::arg3^{+} ura4D18 his3D arg4D4 leu1-32 ade6DN/N*
KFY1569	*h- imr1R (HindIII)::arg3^{+} ura4D18 his3D arg4D4 leu1-32 ade6DN/N*
KFY1597	*h- imr1R (HindIII)::arg3^{+} imr1L (HindIII)::ura4 ura4D18 his3D arg3D4 leu1-32 ade6-210*
KFY1598	*h^{90} imr1R (HindIII)::arg3^{+} imr1L (HindIII)::ura4^{+} ura4D18 his3D arg3D4 leu1-32 ade6-210*
KFY1629	*h- imr1R (HindIII)::arg3^{+} imr1L (HindIII)::ura4^{+} ura4D18 his3D arg3D4 leu1-32 ade6-210*
KFY1703	*h^{+} cdc25-22 leu1-32 ura4D18 arg3D4 his3D*
KFY1871	*h? imr1R (HindIII)::arg3^{+} imr1L (HindIII)::ura4^{+} cdc25-22 ura4D18 his3D arg3D4 leu1-32 ade6-210*
KFY1912	*h- ars1(MluI)::pREP41XCnp1(Leu2+) leu1-32*
