# Supplementary material for: Centromeric Barrier Disruption Leads to Mitotic Defects in Schizosaccharomyces pombe
Source: G3 (Bethesda). 2014 Feb 13;4(4):633–42. doi: 10.1534/g3.114.010397 (PMC4059236; doi:10.1534/g3.114.010397)
Supplement: Supporting Information [file supp_g3.114.010397_TableS2.pdf]

Table S2 List of primers used in this study.

Primer Name	Sequence	Use
47332339	CGGGATCCGGTCGAAAAAAATTCATCCC	amplify *arg3*$^{+}$
47332340	CGGGATCCAAATTGATCCATCCCCTT	amplify *arg3*$^{+}$
5055	GCAAACACATTAATTCTCATGAATTTAGAGATTT<br>CCATTAATAACTTGATTTTAGACTCAATATTAATATTT	Δala
5056	AAATATTAATATTGAGTCTAAAACAAGTTATTAATG<br>GAAATCTCTAAATTCATGAGAATTAATGTGTTTGC	Δala
5057	AAAAAAAAAAAAAAAAGAGATAATTTTTTTATAAGC<br>TACTTTTTATTTTGAAATTAA	Δglu
5058	TTAATTTCAAAATAAAAAGTAGCTTATAAAAAAAT<br>TATCTCTTTTTTTTTTTTTTTT	Δglu
6705	CGCGGATCCATTCGTTFTACCAACTGCT	pcr check
6708	CGCGGATCCTCGCAGCCTTTCAATAACT	pcr check
7453	AATCCAACCGTGAGAAGATGA	Actin RT
7454	ACGACCAGAGGCATACAAAGA	Actin RT
BW84F	CGGCATCGCTTGTACTTTTT	panhet RT
BW84R	GACGGAACCAAATGATGTGA	panhet RT
5373	AAATCACCGGAGCAATGTTT	Site1 RT
5374	AAACACCATGGTTTGTTTGTTA	Site1 RT
5375	TCATTCGTTGTACCAACTGCT	Site2 RT
5376	TGTGTTTGCCATCTTACAATTCA	Site2 RT
201301	TGCGGTTGAGTGTAGGAAAA	Site3 RT
201302	CTGATAGCACATAAACTTTATCATCA	Site3 RT
201307	GCCAGACTTTCTTAGGATATGAATT	cen2het_imr2F
201308	CATCAGTTCGAAATCATTCTACTTG	cen2het_imr2R
201309	TTGTTGCCGCACTTGATG	cen3het_imr3F
201310	TCATGCATACATTACCGATCTACC	cen3het_imr3R
